# Comparative Cytotoxic Evaluation of *Zygophyllum album* Root and Aerial Parts of Different Extracts and Their Biosynthesized Silver Nanoparticles on Lung A549 and Prostate PC-3 Cancer Cell Lines

**DOI:** 10.3390/ph15111334

**Published:** 2022-10-28

**Authors:** Reda F. A. Abdelhameed, Mohamed S. Nafie, Dina M. Hal, Ali M. Nasr, Shady A. Swidan, Maged S. Abdel-Kader, Amany K. Ibrahim, Safwat A. Ahmed, Jihan M. Badr, Enas E. Eltamany

**Affiliations:** 1 Department of Pharmacognosy, Faculty of Pharmacy, Galala University, New Galala 43713, Egypt; 2 Department of Pharmacognosy, Faculty of Pharmacy, Suez Canal University, Ismailia 41522, Egypt; 3 Department of Chemistry, Faculty of Science, Suez Canal University, Ismailia 41522, Egypt; 4 Department of Pharmaceutics, Faculty of Pharmacy, Port Said University, Port Said 42526, Egypt; 5 Department of Pharmaceutics and Industrial Pharmacy, Faculty of Pharmacy, Galala University, New Galala 43713, Egypt; 6 Department of Pharmaceutics and Pharmaceutical Technology, Faculty of Pharmacy, The British University in Egypt, El-Sherouk City 11837, Egypt; 7 The Centre for Drug Research and Development (CDRD), Faculty of Pharmacy, The British University in Egypt, El-Sherouk City 11837, Egypt; 8 Department of Pharmacognosy, College of Pharmacy, Prince Sattam Bin Abdulaziz University, Al-Kharj 11942, Saudi Arabia; 9 Department of Pharmacognosy, Faculty of Pharmacy, Alexandria University, Alexandria 21215, Egypt

**Keywords:** *Zygophyllum album*, total phenolic and flavonoids, antioxidant, UV–VIS, HPLC, AgNPs, TEM, cytotoxicity, PC-3 and A549 cancer cells, apoptosis

## Abstract

The current work demonstrates a comparative study between aerial and root parts of *Zygophyllum album* L. The total phenolic (TPC) and flavonoid content (TFC), in addition to the antioxidant activity, of the crude extracts were investigated, where the aerial parts revealed a higher value overall. By means of UV–VIS and HPLC, rutin and caffeic acid were detected and then quantified as 5.91 and 0.97 mg/g of the plant extract, respectively. Moreover, the biosynthesis of AgNPs utilizing the crude extract of the arial parts and root of *Z. album* L. and the phenolic extracts was achieved in an attempt to enhance the cytotoxicity of the different plant extracts. The prepared AgNPs formulations were characterized by TEM and zeta potential measurements, which revealed that all of the formulated AgNPs were of a small particle diameter and were highly stable. The mean hydrodynamic particle size ranged from 67.11 to 80.04 nm, while the zeta potential ranged from 29.1 to 38.6 mV. Upon biosynthesis of the AgNPs using the extracts, the cytotoxicity of the tested samples was improved, so the polyphenolics AgNPs of the aerial parts exhibited a potent cytotoxicity against lung A549 and prostate PC-3 cancer cells with IC_50_ values of 6.1 and 4.36 µg/mL, respectively, compared with Doxorubicin (IC_50_ values of 6.19 and 5.13 µg/mL, respectively). Regarding the apoptotic activity, polyphenolics AgNPs of the aerial parts induced apoptotic cell death by 4.2-fold in PC-3 and 4.7-fold in A549 cells compared with the untreated control. The mechanism of apoptosis in both cancerous cells appeared to be via the upregulation proapoptotic genes; p53, Bax, caspase 3, 8, and 9, and the downregulation of antiapoptotic gene, Bcl-2. Hence, this formula may serve as a good source for anticancer agents against PC-3 and A549 cells.

## 1. Introduction

Throughout history, natural products have played a prevailing role in the treatment of many diseases. It is proposed that approximately half of all medications presently in use are natural products [1,2]. Nowadays, natural products comprise a large ratio of pharmaceutical products, most particularly in the field of cancer therapy. Examples of plant-derived compounds that are now employed in cancer treatment are paclitaxel, vincristine, irinotecan, and etoposide [3,4]. Generally, natural product research is a tremendous approach for detecting biologically active metabolites with probably unrivaled chemical structures and modes of action [5]. The family *Zygophyllaceae* is a vast plant family. It comprises approximately 27 genera and 285 species among, for which the genus *Zygophyllum* includes 80 species [6]. The genus *Zygophyllum* has been utilized in traditional medical practices for the treatment of miscellaneous diseases such as diabetes, hypertension, rheumatism, and microbial infections [7,8]. Furthermore, numerous biological investigations have proven that plants of the genus *Zygophyllum* exhibited diverse pharmacological activities [7]. For example, the inflammatory [9], cardioprotective [9], gonado-protective [10], and, most importantly, anticancer effects [11] of *Z. album* have been evidenced. From *Z. album,* a variety of phytochemicals have been discovered, including essential oils, triterpenes, sterols, saponins, and polyphenols, specifically flavonoids [10,12,13]. To take the benefits of the natural products, especially from *Z. album,* to the utmost level, nanotechnology offers a great tool for enhancing the therapeutic effect, minimizing the side effect and modulating the therapeutic response of the herbal extracts [14]. Metallic nanoparticles are one of the most promising drug carriers that are attracting the attention of the scientific community. Among metallic nanoparticles, silver nanoparticles have gained increasing interest as they have potential applications in the biomedicine field for their proven anti-angiogenic, antimicrobial, and anticancer activity [15]. Several methods of preparation are involved in the fabrication of silver nanoparticles, but their biosynthesis is considered the optimum method to merge the merits of silver nanoparticles together with those of natural products. Biosynthesis is the synthesis and assembly of nanoparticles with the benefit of green technology to develop a clean, nontoxic, and ecofriendly procedures to prepare nanoparticles [16]. Biosynthesized AgNPs have shown promising cytotoxic effects against different cancer cell lines, including prostate and lung cancer cell lines [17,18]. Based on this, the phytocomponents of *Zygophyllum album* were utilized as capping and reducing agents for the synthesis of green silver nanoparticles, which not only provide stability to these nanoparticles, but also enhance their cytotoxic activity. 

In the current work, we established a comparative study between the aerial parts and root extracts of *Z. album* L. The crude extract and the total phenolic fraction were prepared and tested for the cytotoxic and apoptotic activity against PC-3 and A549 cell lines. Additionally, a silver nanoparticle formula was prepared, and the activity was also recorded, aiming to optimize the biological activity of the tested extracts.

## 2. Results

### 2.1. Total Phenolic and Flavonoid Contents of Z. album L. Roots and Aerial Parts Extracts

*Z. album* L. roots and aerial parts extracts were assessed for their total phenolic and flavonoid contents according to the previously mentioned procedure. The obtained results are shown in Table 1. The outcome of the experiments revealed that the aerial parts extract accumulated a higher percentage of both flavonoids and phenolic compounds compared with the root extract.

### 2.2. In Vitro Antioxidant Activity of Z. album L. Roots and Aerial Parts Extracts

Antioxidants are characterized by their ability to prevent biomolecules, such as nucleic acids, proteins, and polyunsaturated lipids, from undergoing free radical mediated reactions causing oxidative damage. Antioxidants can prohibit oxidizing chain reactions via variable mechanisms, for example the quenching of the reactive oxygen species and metal ions (Fe^2+^ and Cu^+^) chelation. Based on these facts, antioxidants have fruitful effects in the prevention of several diseases where oxidative processes are noteworthy. These include Parkinson’s, Alzheimer’s disease (AD), memory loss, multiple sclerosis, depression, coronary heart disease, aging, and cancer [19,20,21,22].

In this work, three indicative assays (DPPH, FRAP, and TAC) were utilized to evaluate and compare the antioxidant power of *Z. album* L. roots and aerial parts extracts (Table 2). The results showed that *Z. album* L. aerial parts extract revealed a relatively higher antioxidant activity compared with the root extract. As it is well known that flavonoids and phenolic compounds in general possess a powerful antioxidant activity [23], these results could be assigned to the higher contents of flavonoids and phenolics detected in the aerial parts compared with the root extract. 

### 2.3. Quantitative Determination of Caffeic Acid and Rutin Using HPLC

**Figure 1:**
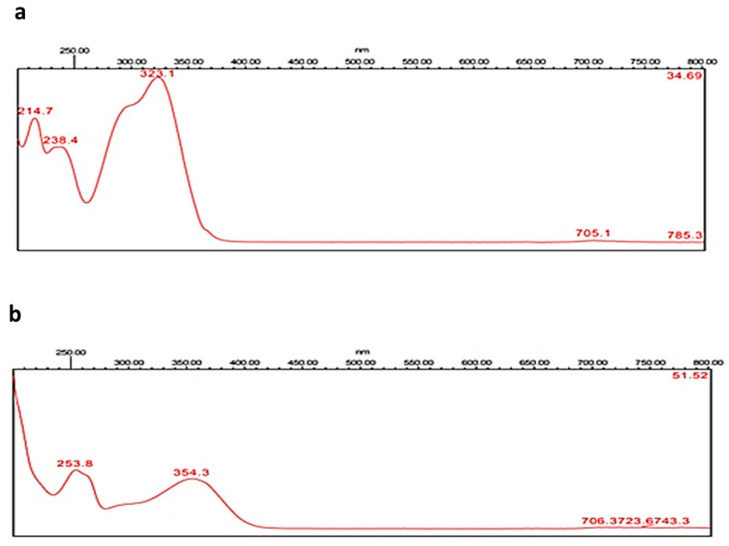
UV–VIS absorbing spectrograms of caffeic acid (**a**) and rutin (**b**).

**Figure 2:**
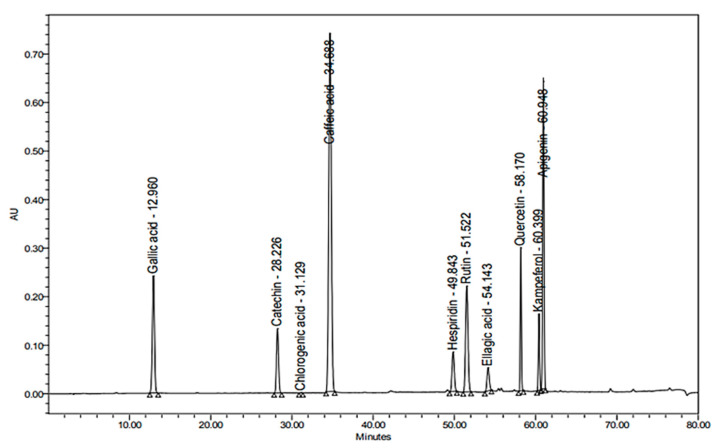
HPLC chromatogram of reference standards detected at λ 280 nm.

**Figure 3:**
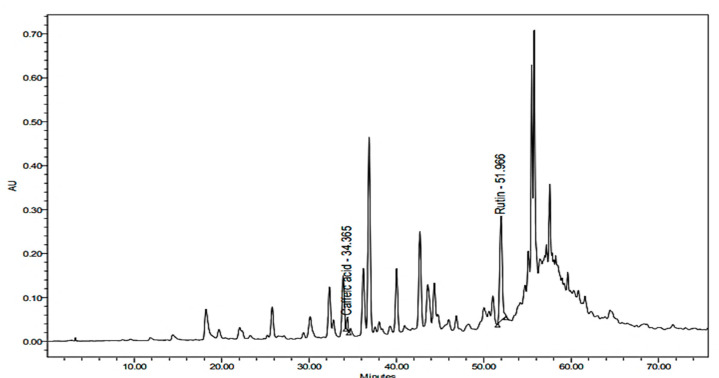
HPLC chromatogram of the methanolic extract of *Z. album* L. (100 mg/mL) detected at λ 280 nm.

Through the application of the proposed method using HPLC, caffeic acid and rutin were quantitatively determined in the methanolic extract of the areal parts of *Z. album*. Accurate identification of the compounds relied on the comparison of the detected retention time, together with the identity of UV spectra of the peaks generated from the plant extract with those of caffeic acid and rutin standards (Figure 1, Figure 2 and Figure 3). 

#### 2.3.1. Method Validation

##### Analytical Solution Stability

In order to guarantee sample stability, the suggested procedure of analysis was performed twice under various conditions including storage at 4 °C for one week and at ambient temperature for 2 days. The results were calculated and compared to those obtained from freshly produced standard solutions and a statistically insignificant difference was found.

##### Linearity

Five different concentrations of caffeic acid and rutin standard solutions were employed to analyze the validity of the suggested procedure. Measurements were achieved in triplicate. With respect to the peak area, a linear correlation was detected over a specific concentration range. The linear regression equations and the correlation coefficient (R^2^) and for caffeic acid and rutin standards were calculated and are illustrated in Table 3.

##### System Precision

To assure the system precision, 10 µL in triplicate of a certain concentration of caffeic acid and rutin standards (100 µg/mL) were applied. Relative standard deviation (%RSD) low values proved the system precision. The data are presented in Table 3. 

##### Method Precision

The methanolic extract solution of *Z. album* (60 µg/mL) was prepared and 10 µL were injected (in triplicate) and analyzed according to the mentioned procedure. %RSD was calculated and is recorded in Table 3. 

##### Limits of Detection and Quantification 

The limits of detection (3 σ/S), as well as the quantification (10 σ/S) of both caffeic acid and rutin were determined (Table 3). The symbol σ represents the standard deviation of the response, while the slope of the calibration curve is symbolized by the letter S.

##### Analysis of *Z. album* Extract 

The proposed method was employed to estimate the caffeic acid and rutin concentrations in the *Z. album* methanolic extract. Based on the obtained regression equations, the concentrations were calculated. Measurements were achieved four times. Caffeic acid and rutin were determined as 0.97 ± 0.000168 and 5.91 ± 0.000371 mg/g of the plant extract, respectively. 

### 2.4. Characterization of Silver Nanoparticles Formulae

**Figure 4:**
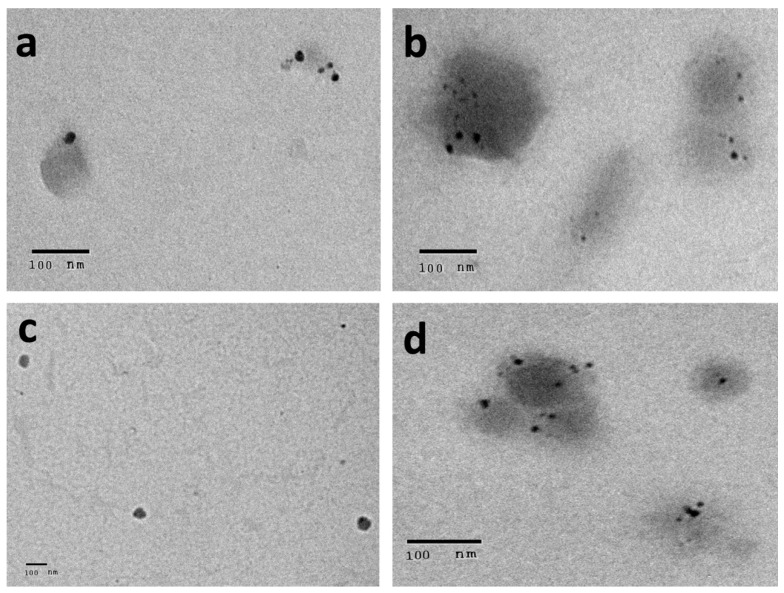
Transmission electron microscope images for AgNPs of (**a**) crude extract of aerial parts, (**b**) crude extract of root, (**c**) phenolic extract of the aerial parts, and (**d**) phenolic extract of the root of *Z. album* L.

**Figure 5:**
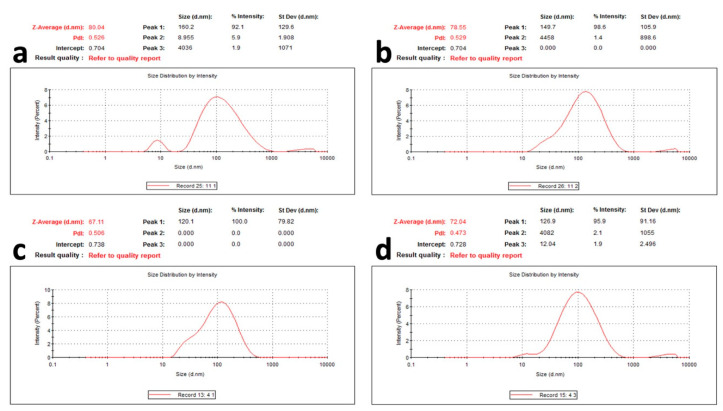
Particle size and particle size distribution of AgNPs of (**a**) crude extract of aerial parts, (**b**) crude extract of root, (**c**) phenolic extract of the aerial parts, and (**d**) phenolic extract of the root of *Z. album* L.

The prepared AgNPs of all of the extracts showed a yellowish-brown colour. The morphological characteristics of the prepared AgNPs related to the shape and size distribution were elucidated using TEM and were confirmed by dynamic light scattering technique. From the TEM images, the nanoparticles exhibited a spherical, uniform in size, and monodisperse appearance (Figure 4). From the images, very small nanoparticles less than 20 nm in size were obtained for all of the prepared extracts. With more detailed examination, the particles appeared to be surrounded by a thin layer. This suggested the presence of a non-metallic organic capping agent that stabilizes the particles. A similar observation was found by Gengana and coworkers [18]. DLS utilizes the scattering of radiation through its interaction with a sample. The scattered light intensity fluctuation was used to determine the particle hydrodynamic diameter [24]. As seen in Figure 5, the mean hydrodynamic diameter measured by DLS ranged from 67.11 to 80.04 nm for the AgNPs of the phenolic extract of the aerial parts and the crude extract of the the aerial parts of *Z. album,* respectively. A similar mean diameter range was obtained in different studies [25,26]. Interestingly, a higher mean particle size was observed when measuring the particle size using the DLS technique.

The difference in the mean diameter between TEM and DLS is related to the variation in the principals of measurements between the two techniques [18]. The same observation was found by Ameen et al., who explained the difference in the techniques as the TEM being a local analysis of particles, while DLS is a cumulative analysis of light scattering in an aqueous medium, this makes the two techniques fundamentally different and they are based on the dry diameter of particles and the hydrodynamic diameter, respectively [27]. It is also noticed that the PDI results were around 0.5, which is moderate polydispersity for the prepared nanoparticles from all of the extracts. Although the PDI value was relatively high, a higher PDI value of biosynthesized AgNPs was reported by Kartini et al., who stated that PDI > 0.7 is considered polydisperse [28]. Similar values were obtained by Khan et al. and Shahzad et al. [29,30].

**Figure 6:**
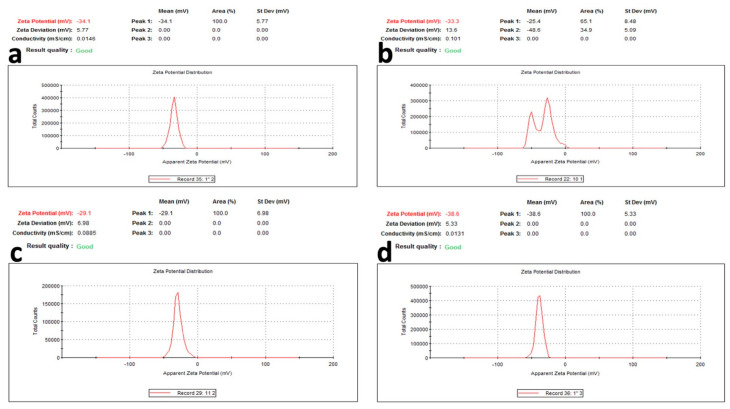
Zeta potential of AgNPs of (**a**) crude extract of aerial parts, (**b**) crude extract of root, (**c**) phenolic extract of the aerial parts, and (**d**) phenolic extract of the root of *Z. album* L.

For surface charge, which is an indication of the stability of the colloidal systems, it has been reported that when the zeta potential is 30 mV, this indicates a highly stable colloidal system. From Figure 6, the zeta potential ranged from 29.1 to 38.6 mV for the phenolic extract of the aerial parts and phenolic extract of the root of *Z. album,* respectively. These values reflect the stability of the prepared AgNPs for all of the extracts, where the AgNPs of the phenolic extracts of the root showed the most stability.

### 2.5. Cytotoxic Activity

#### 2.5.1. MTT Assay

**Figure 7:**
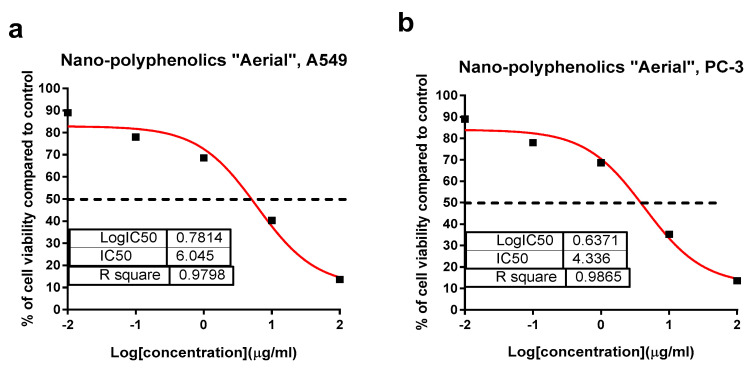
Percentage of cell viability vs. log [con. µg/mL], R square ≈ 1 using the GraphPad prism software. (**a**) Cytotoxicity of the AgNPs formula of polyphenolics aerial extract against A549 cells. (**b**) Cytotoxicity of the AgNPs formula of the polyphenolics aerial extract against PC-3 cells using an MTT assay.

Crude and polyphenolic extracts of the root and aerial parts of *Z. album* L. were screened for their cytotoxicity against PC-3 and A549 cell lines using an MTT assay. As summarized in Table 4, the polyphenolic extract of the aerial parts exhibited a promising cytotoxicity against A549 and PC-3 with IC_50_ values of 11.4 and 13.4 µg/mL, while others exhibited a moderate to week cytotoxicity. These results agreed with previous studies on *Z. album* L., which reported an interesting anticancer capacity against human lung carcinoma (A-549) and colon adenocarcinoma (DLD-1) cells (IC_50_ = 37 and 48 µg/mL, respectively) [31]. Upon preparing the AgNPs formula, the cytotoxic activities of the tested samples were improved. The AgNPs formula of the polyphenolics portion of the aerial parts exhibited a potent cytotoxicity with IC_50_ values of 6.1 and 4.36 µg/mL compared with Doxorubicin with IC_50_ values of 6.19 and 5.13 µg/mL against A549 and PC-3, respectively (Figure 7). Similarly, the nano formulations of the polyphenolic portion of *Z. album* roots, as well as the crude extracts of both plant parts exhibited an enhanced cytotoxicity with promising IC_50_ values ranging 11.7–13.1 µg/mL comparted with that in the normal form. 

#### 2.5.2. Apoptosis-Induction Activity

##### Annexin V/PI Staining

**Figure 8:**
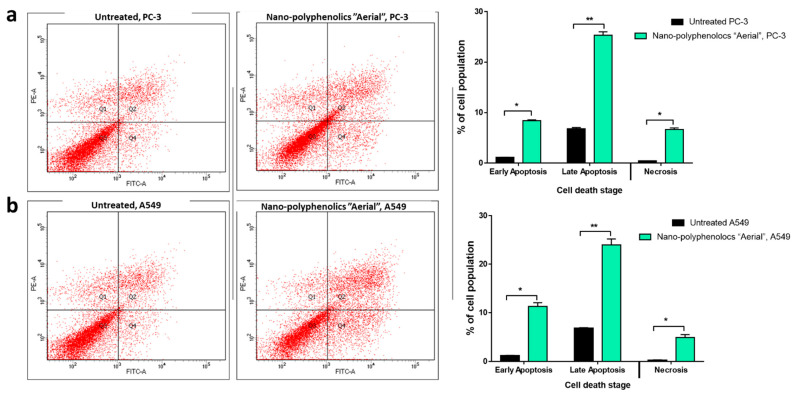
Cytograms and bar representation for apoptosis-necrosis assessment using flow cytometry. (**a**) Annexin V/PI staining of untreated and treated PC-3 cancer cells with the AgNPs formula of the polyphenolics portion of the aerial parts (IC_50_ = 4.36 µg/mL, 48 h). (**b**) Annexin V/PI staining of untreated and treated A549 cancer cells with the AgNPs formula of the polyphenolics portion of the aerial parts (IC_50_ = 6.1 µg/mL, 48 h). Q1: Necrosis, Q2: Late apoptosis, Q4: Early apoptosis. Lower panel. * (*p* ≤ 0.05) and ** (*p* ≤ 0.001) significantly different using the unpaired test in GraphPad prism.

Annexin V/PI staining was used to investigate the mechanism of apoptosis induction through thr AgNPs formula of the polyphenolics portion of the aerial parts on PC-3 and A549 cells. Compared with the untreated control cells, the nano-formula caused a 33.74% increase in total prostate apoptotic cell death (Figure 8) by 8.37% early and 25.37% late apoptosis. As a result, it caused a 4.2-fold increase in PC-3 cell apoptosis. In addition, 35.3% of the untreated control cells died from apoptosis, whereas 7.48% of the treated control cells did. As a result, it caused a 4.7-fold increase in apoptosis in A549 cells. Consequently, the AgNPs formula of the polyphenolics portion of the aerial parts exhibited apoptosis in PC-3, and A549 cells with nearly the same ratio.

##### Gene Expression Analysis of Apoptosis-Related Genes Using RT-PCR

The apoptotic mechanism in PC-3 and A549 cancer cells induced by the formulated AgNPs of *Z. album* aerial parts polyphenolics in PC-3 and A549 cells was inspected using the RT-PCR technique. The expressions of apoptosis-related genes P53, Bax, Caspases 3 and 9, and Bcl-2 were analyzed in treated and untreated cells. Table 5 summarizes the effects of AgNPs of the aerial polyphenolics on PC-3 and A549 cells. Treatment with the nano formula in PC-3 and A549 cells caused expression upregulation of proapoptotic genes; p53 by 9.06 and 11.03-fold, Bax by 6.54 and 8.63-fold, Caspase-3 by 12.3 and 9.0-fold, and Caspase-9 by 10.1 and 8.1-fold, respectively. These effects in PC-3 and A549 were accompanied by the downregulation of the anti-apoptotic gene; Bcl-2 by 0.31 and 0.24-fold, respectively. Therefore, apoptosis was found to be induced in both cancer cell lines via mitochondrial-mediated apoptosis.

Additionally, as Caspase-8 (CASP8) is a cysteine protease and plays a pivotal role in the extrinsic apoptotic signaling pathway via death receptors, the extrinsic activity was investigated. AgNPs of the aerial polyphenolics upregulated caspase-8 gene expression by 2.13-fold in PC-3 cells, while it upregulated caspase-8 gene expression by 4.16-fold in A549 cells. Hence, the extrinsic activity was also activated, but the intrinsic one was favored as the effective cell death mechanism. 

## 3. Materials and Methods

### 3.1. General Experimental Procedures

Absorbance measurements were recorded using a UV–visible spectrophotometer (Milton Roy, Spectronic 1201, Houston, TX, USA) for the determination of the total phenolic (TPC) and total flavonoid (TFC) contents in the *Z. album* extract, as well as its in vitro antioxidant activities (DPPH, FRAP, and TAC).

### 3.2. Plant Material

*Z. album* L. was collected from Marsa Matrouh at the Northern Coast of the Mediterranean in Egypt during May 2019. It was identified at the Faculty of Science, Alexandria University. In the herbarium of the Pharmacognosy Department, Faculty of Pharmacy, Suez Canal University, Ismailia, Egypt, a voucher specimen of the plant (#ZA-2019) was kept. 

### 3.3. Extraction and Isolation

For the preparation of the extracts, 900 g and 1.8 kg of the chopped small pieces of roots and aerial parts of *Z. album,* respectively, were separately macerated in (2 L) methanol at room temperature three times. The combined extracts were dried under reduced pressure to give viscous crude extract (30 g and 50 g of the roots and areal parts extracts respectively). For preparing the phenolic portion, 2 gm of different extracts were treated with an aqueous solution of 5% sodium carbonate (100 mL) for one hour with the aid of sonication. The extract was filtered and then rinsed with distilled water. [32]. The aqueous solution was partitioned with *n*-butanol to extract a non-phenolic portion. The remaining aqueous solution was neutralized by a hydrochloric acid using litmus paper as an indicator. Finally, the phenolic portion was extracted by using ethyl acetate three times through liquid–liquid extraction and was concentrated under a vacuum. 

### 3.4. Total Phenolic Content Assay

Using the Folin–Ciocalteu spectrophotometric method, the total phenolic content (TPC) in *Z. album* crude extracts was determined [33,34]. In this method, a mixture of 10% Folin–Ciocalteau (3 mL), the plant extract (0.05 mL), and 7.5% sodium bicarbonate (0.8 mL) was prepared and then kept at 25 °C (30 min). Then, the UV absorbance at λ 765 nm was recorded in triplicate. The TPC was expressed as mg gallic acid equivalents (GAE)/g extract.

### 3.5. Total Flavonoid Content Assay

The total flavonoid content (TFC) in the *Z. album* roots and aerial parts crude extracts was quantified using the AlCl_3_ spectrophotometric method mentioned in [35]. In short, a mixture of the extract (0.1 mL), distilled water (3.90 mL), and 5% NaNO_2_ solution (0.3 mL) was prepared and left to react for 5 min. Then, 10% AlCl_3_ solution (0.3 mL) was added. The mixture was given 6 min to continue reacting. After that, 1 mM^−1^ NaOH (2 mL) was added to the mixed solution. All of the samples received 2.4 mL of distilled water at the end. Finally, the UV absorbance was recorded at λ 510 nm in triplicate against a sample blank without reaction. TFC is represented as mg quercetin equivalents (QE)/g extract.

### 3.6. In Vitro Antioxidant Activity 

#### 3.6.1. DPPH Radical Scavenging Activity

According to the procedure described in [36], a solution of 2,2-diphenyl-1-picrylhydrazyl (DPPH) radical was freshly produced in methanol with a concentration of 0.004% *w*/*v,* and then kept in the dark at 10 °C. The methanolic solutions of *Z. album* crude extracts were prepared. Then, an aliquot of the prepared sample solution (40 µL) was mixed with 3 mL of the DPPH solution. The immediate UV absorbance measurements λ 515 nm were taken. Then, data were recorded at intervals of one minute for 16 min to monitor the decline in absorbance until the absorbance stabilised. The absorbances of the control and ascorbic acid (the reference compound) were estimated. All of the determinations were achieved in triplicate and then averaged. Employing the following equation:

PI = [(AC − AT)/AC × 100]

The DPPH radical scavenging potential was estimated. Where PI = the DPPH radical’s percentage inhibition, AC = absorbance of the control at t = 0 min, and AT = absorbance of the sample + DPPH at time = 16 min. From the dose response curve, the IC_50_ of the extract, required to inhibit the DPPH radical by 50%, was calculated.

#### 3.6.2. Ferric Reducing Antioxidant Power Assay

The Ferric reducing power assay (FRAP) of the extracts was estimated spectrophotometrically using the previously described procedure [37,38]. This method relied on the proportional reduction of ferricyanide ions to various test sample concentrations. In a nutshell, 2.5 mL of sodium phosphate buffer (0.2 M, pH = 6.6) and 1% *w*/*v* K_3_[Fe (CN)_6_] solution were combined with 1 mL of the extract’s methanolic solution. The reaction was maintained for 20 min at 50 °C before being acidified with 10% *w*/*v* trichloroacetic acid (2.5 mL), and then centrifuged for 10 min at 650 rpm. Then, the produced supernatant was mixed with 0.5 mL of 0.1% *w*/*v* FeCl_3_ solution (freshly prepared in deionized water). The UV absorbance of the reaction mixture was measured at λ 700 nm. The outcomes were presented as m Mol Fe^+2^ equivalent/g dry sample and butyl hydroxy toluene (BHT) was used as a standard.

#### 3.6.3. Total Antioxidant Capacity (TAC) Assay

Using a phosphomolybdenum spectrophotometric assay [39,40], the total antioxidant capacities (TAC) of the *Z. album* roots and aerial parts crude extracts were determined. This procedure relied on the conversion of Mo^+6^ to Mo^+5^ by an antioxidant under acidic conditions, resulting in the formation of the green coloured phosphate/Mo^+5^ complex. In this method, the extract was dissolved in methanol then an aliquot of 0.2 mL of the prepared solution was added to 0.1 mL of the reagent, which is composed of 2 mM Na_3_PO_4_, 4 mM (NH_4_)_2_MoO_4_, and 0.6 M H_2_SO_4_. Following a 90-min incubation period at 95 °C, the reaction mixture was cooled, and then its UV absorbance at λ 695 nm was recorded. Using the gallic acid standard curve, the outcomes were expressed as mg equivalents of gallic per g of extract (mg GAE/g). Ascorbic acid was utilized as an authentic.

### 3.7. HPLC-DAD Quantitative Analysis

#### 3.7.1. Authentic Standard Compounds

Ten of the phenolic and flavonoids that are of common occurrence in plants were used for the qualitative analysis of *Z. album* L. extract (namely: apigenin, kaempferol, quercetin, rutin, hesperidin, catechin, beside the four acids: ellagic, caffeic, chlorogenic, and gallic acid). Two of them were detected, namely: caffeic acid and rutin. These two compounds were purchased from Nawah Scientific, Egypt, and were used as reference compounds, their purity was certified to be 99%. The two authentic compounds were used for quantitative analysis of the aerial parts extract of *Z. album* L. 

#### 3.7.2. Instrumentation

Analysis was performed utilizing HPLC (Waters 2690 Alliance HPLC system coupled with Waters 996 photodiode array detector). Analysis was performed using C18 Inertsil ODS column with dimensions of 4.6 × 250 mm and 5 µm particle size. 

#### 3.7.3. Operating Conditions

The combined methanolic solution of the caffeic acid and rutin standards was prepared. The injected volume was 10 µL. Then, 0.1% solution of H_3_PO_4_ in H_2_O:CH_3_CN was utilized as the mobile phase (pH = 3.5). The flow rate was maintained at 1 mL/min. The gradient technique was applied as follows; first: 5 min of acidified H_2_O: CH_3_CN (95:5, *v*/*v*) was used, second: 30 min increasing the concentration of CH_3_CN to 80%, third: a plateau period (25 min). Finally, the CH_3_CN concentration was gently reduced to 10%. During the last 5 min, isocratic elution was used. Measurement of the absorbance was achieved at λ 280 nm [41,42].

#### 3.7.4. Calibration Graphs

Caffeic acid and rutin stock solutions were produced by dissolving 100 mg of each in methanol (25 mL). The obtained solutions were filtered using a 0.22 µm syringe filter. The filtrate was quantitatively transferred to a volumetric flask (100 mL). Methanol was used for volume adjustment. The stock solutions were tightly closed then kept in the refrigerator. Serial dilutions were prepared and used to construct the calibration curves by injection of an aliquot of 10 µL from each concentration. The peak areas were recorded and plotted against their corresponding concentrations. 

#### 3.7.5. Sample Preparation

The extract solution of *Z. album* L. was obtained by precisely weighing 1 g of the extract then dissolving it in methanol (50 mL), followed by gentle sonication for 15 min. The produced solution was filtered through a 0.22 µm Nylon syringe filter. The filtrate was quantitatively transferred to a volumetric flask (100 mL). Methanol was used for volume adjustment. After dilution, a solution with a final concentration of 60 mg/100 mL was obtained, of which 10 µL was injected.

### 3.8. Preparation and Characterization of Nanoparticles

#### 3.8.1. Biosynthesis of Silver Nanoparticles

The synthesis of AgNPs in the presence of aerial parts and root extracts of *Z. album* L. and the total phenolic fraction were prepared using a modified method of that reported by [43,44]. First, 10 mg of the extract was dissolved in 1 mL of ethanol, then it was added to 10 mL of 10 mM AgNO_3_. A few drops of 1 M NaOH were added, and the mixture was agitated for 1 h at 400 rpm at 60 °C in the dark. All of the prepared nanoparticles were purified by centrifugation at 15,000 rpm for 1 h at 4 °C. The AgNPs were re-dispersed in double-distilled water and sonicated for 30 s in a sonicating water bath, then centrifuged under the same previous conditions. The washing protocol using double-distilled water was repeated three times and the obtained silver nanoparticles were stored for further characterization.

#### 3.8.2. Characterization of Silver Nanoparticles 

The prepared silver nanoparticles were characterized using transmission electron microscopy and zeta potential. In brief, microscopy (TEM) was carried out to examine the size and surface morphology of the synthesized AgNPs. The sample preparations were further diluted 50 times with double distilled water. Then, the diluted samples were negatively stained with phosphotungstic acid and dried on carbon-coated copper grids. The thin film formed was air-dried at room temperature and observed using transmission electron microscope (JTEM model 1010, JEOL^®^, Tokyo, Japan) with a voltage of 80 kV. The hydrodynamic mean diameter was measured using the DLS technique with a Zetasizer (Nano ZS, Malvern Instruments Ltd., Malvern, UK). Before analysis, each sample was diluted 20 times with distilled water. All of the measurements were carried out in triplicate at room temperature (25 °C). The surface charge of the nanoparticles is of high importance as it indicates the stability of the colloidal system. The zeta potential of the prepared AgNPs was measured with the same procedures as the hydrodynamic mean diameter using a zetasizer.

### 3.9. Cytotoxic Activity

#### 3.9.1. MTT Assay

Lung (A549) and prostate (PC-3) cancer cell lines were obtained from the National Cancer Institute in Cairo, Egypt, cultured on Dulbecco’s Modified Eagle Medium and Roswell Park Memorial Institute Medium (RPMI-1640/DMEM) supplemented with L-glutamine (Lonza Verviers SPRL, Verviers, Belgium, cat#12-604F). The cells were grown in a medium containing 10% fetal bovine serum (FBS; Sigma-Aldrich, St. Louis, MO, USA) and 1% penicillin–streptomycin (Lonza, Verviers, Belgium). Each batch of cells was kept in an incubator at 37 degrees Celsius and 5% carbon dioxide (NuAire). At a density of 5 × 104, the cells were plated in triplicate in a 96-well plate. Then, they were exposed to various concentrations of the tested samples on the second day. Cell viability was assessed using the MTT solution (Promega, Madison, WI, USA) [45]. This plate was incubated for 3 h. Subsequently, an ELISA microplate reader was used to assess the absorbance level (BIO-RAD, model iMark, Tokyo, Japan). The IC_50_ values were generated using GraphPad Prism 7, in the same way as was previously reported [32,46].

#### 3.9.2. Investigation of Apoptosis 

##### Annexin V/PI Staining and Cell Cycle Analysis 

Both PC-3 and A549 cells were incubated into six-well culture plates (3–5 × 10^5^ cells/well) overnight, and they were then treated with the sample at a dose equal to its determined IC_50_. Then, media supernatants and cells were collected, and the cells were suspended in 100 µL of Annexin binding buffer solution 25 mM CaCl2, 1.4 M NaCl, and 0.1 M Hepes/NaOH, pH 7.4 and incubation with Annexin V-FITC solution (1:100) and propidium iodide (PI) at a concentration equals 10 µg/mL in the dark for 30 min. Cytoflex FACS cells were employed to collect the stained samples, and cytExpert was used for the data analysis [47,48].

##### Gene Expression Analysis (RT-PCR) for the Selected Genes

The expression of pro-apoptotic genes (P53, Bax, and Caspases-3, 8, and 9) and anti-apoptotic genes (Bcl-2) were assessed to investigate the apoptotic pathway (Table 6, forward and reverse sequencing). PC-3 and A549 cells were treated with AgNPs of *Z. album* aerial parts polyphenolics at a dose equal to their IC_50_ value (4.36 and 6.1 µg/mL, respectively), and then incubated for 48 h. Subsequently, RT-PCR reactions were run as per protocol, with data presented as cycle thresholds (Ct) and ΔΔ Ct for estimating the relative abundance of each gene relative to the housekeeping gene (β-actin), as previously described [49,50,51].

## 4. Conclusions

Herein, the crude and polyphenolic extracts from *Z. album* roots and aerial parts were prepared. The TPC, TFC, and in vitro antioxidant activity of *Z. album* roots and aerial parts were estimated, and the obtained results revealed that the plant aerial parts possessed a notable antioxidant activity, which could be attributed to its high contents of phenolic and flavonoids. Furthermore, biosynthesized AgNPs were successfully prepared using crude and polyphenolic extracts of the aerial parts and root of *Z. album.* AgNPs obtained from all of the extracts, which were of a small particle size and the colloidal dispersions were considered stable with a high surface charge. Furthermore, AgNPs synthesized from all extracts enhanced their cytotoxic activities and it was found that the AgNPs formula of the polyphenolics portion of the aerial parts exhibited a potent cytotoxicity with IC_50_ values of 6.1 and 4.36 µg/mL against A549 and PC-3, respectively, compared with Doxorubicin with IC_50_ values of 6.19 and 5.13 µg/mL, respectively. It increased prostate and lung cell death by nearly four-fold compared with the untreated cells. RT-PCR analysis revealed that the AgNPs of the polyphenolics of *Z. album* aerial parts mediated its apoptotic effects in PC-3 and A549 cells via both extrinsic and intrinsic pathways through the upregulation of proapoptotic genes and the downregulation of the anti-apoptotic genes. Hence, the AgNPs formula of the polyphenolics aerial extract of *Z. album* L. may serve as a good source for anticancer agents against PC-3 and A549 cells. 

## Figures and Tables

**Table 1:** Total phenolic and flavonoid contents of *Z. album* L. roots and aerial parts extracts.

Sample Code	Total Flavonoids (mg/gm)	Total Phenolic (mg/gm)
*Z. album* L. aerial parts extract	2.33 ± 0.51	5.26 ± 0.48
*Z. album* L. roots extract	1.36 ± 0.22	3.86± 0.62

**Table 2:** Antioxidant activities of *Z. album* L. roots and aerial parts extracts.

Sample	DPPH (IC_50_ in µg/mL)	FRAP (mMol Fe^+2^/g)	TAC (mg GAE/g)
*Z. album* L. aerial parts extract	291.9 ± 2.8	1.38 ± 0.51	37.82 ± 1.94
*Z. album* L. roots extract	541.9 ± 36.2	0.93 ± 0.35	20.47 ± 1.35
Ascorbic acid	10.6 ± 0.8	--	--
BHT	--	6.98 ± 0.76	76.43 ± 3.89

**Table 3:** Validation parameters of the HPLC method for simultaneous quantitative analysis of caffeic acid and rutin in the methanolic extract of *Z. album* L.

Validation Parameter	Caffeic Acid	Rutin
Linearity range (µg/mL)	15–150	10–200
Regression equation	y = 43,286.51 × −51,445.4	y = 11,062.25 × −24,842.13
Correlation coefficient (R^2^)	0.990	0.995
System precision (%RSD)	0.36	0.59
Method precision (%RSD)	1.28	1.69
Limit of detection (µg/mL)	0.5	0.7
Limit of quantification (µg/mL)	1.8	2.4

Scanned at λ = 280 nm, %RSD: relative standard deviation.

**Table 4:** Summarized IC_50_ values of crude and polyphenolic extracts of root and aerial parts of *Z. album* L. in both the normal and AgNPs formulae.

	Sample	Working Concentration	IC_50_ (µg/mL) *	
			A549	PC-3
Normal	Crude root extract	0.1, 1, 10, 50, 100 µg/mL	≥50	42.1 ± 1.9
	Polyphenolics root extract		29.8 ± 0.89	26.7 ± 1.01
	Crude aerial extract		27.1 ± 0.8	22.7 ± 1.0
	Polyphenolics aerial extract		11.4 ± 0.84	13.4 ± 0.87
AgNPs	Crude root extract		42.6 ± 2.05	36.2 ± 2.0
	Polyphenolics root extract		13.1 ± 0.97	11.7 ± 0.93
	Crude aerial extract		12.4 ± 0.68	21.7 ± 0.97
	Polyphenolics aerial extract		6.1 ± 0.13	4.36 ± 0.12
Reference drug	Doxorubicin		6.19 ± 0.58	5.13 ± 0.64

* IC_50_ values are expressed as mean ± SD of three independent trials and were calculated by non-linear regression curve fit using GraphPad prism.

**Table 5:** Gene expression of the apoptosis-related genes in PC-3 and A549 cells treated with the AgNPs formula of the polyphenolics portion of the aerial parts (IC_50_ = 4.36 µM, 48 h).

Treated Cells	Apoptosis-Genes	Genes	2^−ΔΔCt^ (Fold Change ±SD) ^#^
Treated-PC3 cells	Anti-apoptotic gene	Bcl-2	0.31 ± 0.01
	Pro-apoptotic genes	P53	9.06 ± 0.74
		Bax	6.54 ± 0.71
		Caspase-3	12.3 ± 1.09
		Caspase-8	2.13 ± 0.12
		Caspase-9	10.1 ± 1.03
Treated-A549 cells	Anti-apoptotic gene	Bcl-2	0.24 ± 0.01
	Pro-apoptotic genes	P53	11.3 ± 0.67
		Bax	8.63 ± 0.73
		Caspase-3	9.3 ± 0.87
		Caspase-8	4.16 ± 0.32
		Caspase-9	8.1 ± 1.0

^#^ Values are expressed as mean ± SD for three independent experimental runs. The housekeeping gene is β-actin. ΔΔCt: the difference between the mean values of the gene CT values in the treated and control groups. Fold of change in the untreated control = 1.

**Table 6:** Sequences of forward and reverse primers.

Gene	Forward	Reverse
P53	5′-CCCCTCCTGGCCCCTGTCATCTTC-3′	5′-GCAGCGCCTCACAACCTCCGTCAT-3′
Bax	5′-GTTTCATCCAGGATCGAGCAG-3′	5′-CATCTTCTTCCAGATGGTGA-3′
CASP-3	5′-TGGCCCTGAAATACGAAGTC-3′	5′-GGCAGTAGTCGACTCTGAAG-3′
CASP-8	5′-AATGTTGGAGGAAAGCAAT-3′	5′-CATAGTCGTTGATTATCTTCAGC-3′
CASP-9	5′-CGAACTAACAGGCAAGCAGC-3′	5′-ACCTCACCAAATCCTCCAGAAC-3′
Bcl-2	5′-CCTGTGGATGACTGAGTACC-3′	5′-GAGACAGCCAGGAGAAATCA-3′
β-actin	5′-GTGACATCCACACCCAGAGG-3′	5′-ACAGGATGTCAAAACTGCCC-3′

## Data Availability

Data is contained within the article.
